# Tumoricidal activity of low-energy 160-KV versus 6-MV X-rays against platinum-sensitized F98 glioma cells

**DOI:** 10.1093/jrr/rru084

**Published:** 2014-09-28

**Authors:** Sara N. Lim, Anil K. Pradhan, Rolf F. Barth, Sultana N. Nahar, Robin J. Nakkula, Weilian Yang, Alycia M. Palmer, Claudia Turro, Michael Weldon, Erica Hlavin Bell, Xiaokui Mo

**Affiliations:** 1 Biophysics Graduate Program, The Ohio State University, 113 Biological Sciences Building, 484 W 12th Avenue, Columbus, OH 43210, USA; 2 Department of Astronomy, The Ohio State University, 4055 McPherson Laboratory, 140 W 18th Avenue, Columbus, OH 43210, USA; 3 Department of Pathology, The Ohio State University, 4132 Graves Hall, 333 West 10th Avenue, Columbus, OH 43210, USA; 4 Department of Chemistry and Biochemistry, The Ohio State University, Newman & Wolfrom Laboratory, 100 W 18th Avenue, OH 43210, USA; 5 Department of Radiation Oncology, The Ohio State University, 300 W 10th Avenue, Columbus, OH 43210, USA; 6 Center for Biostatistics, The Ohio State University, 2012 Kenny Road, Columbus, OH 43210, USA

**Keywords:** comparison of 160-kV vs 6-MV X-rays, platinum radiosensitization, F98 rat glioma, B16 murine melanoma, dose enhancement factor

## Abstract

The purposes of this study were (i) to investigate the differences in effects between 160-kV low-energy and 6-MV high-energy X-rays, both by computational analysis and *in vitro* studies; (ii) to determine the effects of each on platinum-sensitized F98 rat glioma and murine B16 melanoma cells; and (iii) to describe the *in vitro* cytotoxicity and *in vivo* toxicity of a Pt(II) terpyridine platinum (Typ-Pt) complex. Simulations were performed using the Monte Carlo code Geant4 to determine enhancement in absorption of low- versus high-energy X-rays by Pt and to determine dose enhancement factors (DEFs) for a Pt-sensitized tumor phantom. *In vitro* studies were carried out using Typ-Pt and again with carboplatin due to the unexpected *in vivo* toxicity of Typ-Pt. Cell survival was determined using clonogenic assays. In agreement with computations and simulations, *in vitro* data showed up to one log unit reduction in surviving fractions (SFs) of cells treated with 1–4 µg/ml of Typ-Pt and irradiated with 160-kV versus 6-MV X-rays. DEFs showed radiosensitization in the 50–200 keV range, which fell to approximate unity at higher energies, suggesting marginal interactions at MeV energies. Cells sensitized with 1–5 or 7 µg/ml of carboplatin and then irradiated also showed a significant decrease (*P* < 0.05) in SFs. However, it was unlikely this was due to increased interactions. Theoretical and *in vitro* studies presented here demonstrated that the tumoricidal activity of low-energy X-rays was greater than that of high-energy X-rays against Pt-sensitized tumor cells. Determining whether radiosensitization is a function of increased interactions will require additional studies.

## INTRODUCTION

High-energy linear accelerators (LINACs) are in widespread use for clinical radiation therapy. Typically, 6–15-MV LINACs generate Bremsstrahlung radiation with mean energies ranging from a few MeV up to a maximum, corresponding to the peak energy of the accelerating voltage. Megavoltage (MV) radiation has been used clinically due to its much greater depth of penetration compared with low-energy X-rays in the 100–250 kV range. The therapeutic efficacy of high versus low-to-medium energy (E < 100 keV) X-rays in combination with radiosensitization by high atomic number elements, (high-Z or HZ), such as Au [1–16] and to a much lesser extent Pt [12, 17, 18], has been of great interest. At lower energies, the cross-sections for atomic inner-shell photoionization, and hence the number of ejected tumoricidal Auger electrons, are much higher [16]. This dichotomy between the radiobiological effects of high- versus low-energy X-rays in the presence of HZ sensitizers for radiation therapy has generated considerable interest in recent years, including the role of broadband and monochromatic X-ray sources, where k/MV and k/MeV refer, respectively, to broadband and monoenergetic X-rays [12, 13, 16].

A number of studies have been carried out at the European Synchrotron Radiation Facility (ESRF) by Elleaume and her research team [18–21] and Barth and his co-workers at The Ohio State University (OSU) [22, 23] comparing the radiobiologicaleffects and therapeutic efficacy of high-energy 6-MV X-rays in combination with cisplatin or carboplatin versus those obtained using monoenergetic synchrotron X-rays at much lower-keV energies [18, 19]. Other studies have focused on gold (Z = 79) nanoparticles (GNP) [1–11], which are non-toxic and interact efficiently with X-rays. However, some Monte Carlo simulations have shown that the concentration of GNPs required for irradiation with 6-MV X-rays would not be clinically achievable [9]. Recently, Leung *et al*. [8] concluded that irradiation of GNPs with lower-energy X-rays would be more effective for cell killing. Studies on endothelial cell damage and analysis of the effects as a function of photon energies from 100 keV to 1 MeV demonstrated that it was mostly the E < 100 keV portion of the 6-MV spectrum that had significant dose enhancement factors (DEF) [7]. Other investigators have studied cell-specific energy deposition in proximity to the irradiated GNPs, and have suggested that this could be a possible biological mechanism for their radiosensitizing properties [6, 11], which might make them a useful therapeutic modality [5].

The broad peak of the Bremsstrahlung spectral distribution is ∼80 keV from a 250-kV source, which is close to the K-ionization edge of Au and Pt. This also encompasses lower energies towards the L-edge of Pt at ∼14 keV. This observation provides the physical basis of HZ radiosensitization by lower-energy X-rays via Auger breakdown and electron cascades following inner-shell ionizations. In previous reports [12–14] we have described the theoretical interaction of monochromatic keV X-rays and MeV photons with HZ elements and the ensuing photon and electron emissions, together with new pathways that might be implemented to enhance Auger decays. In the present report, we have studied in more detail the specific energy dependence of photoionization-induced Auger electron–photon cascades.

Primary X-ray photons at each energy in the broadband 160-KV or 6-MV sources, up to 160 keV and 6 MeV respectively, produce a spectrum of secondary electrons and photons due to Auger cascades within the HZ-sensitized tumor cells. These secondary electrons and photons are then attenuated as a function of depth in tissue up to their characteristic effective ranges. In an earlier Monte Carlo [12] study using Geant4 on Au atoms, we reported the mean photon energies and the number of electrons emitted (corresponding to different incident energies) and then deposited as a function of depth from 0 to 15 cm in a water phantom. There was significant enhancement of the secondary photon energy deposited, but not a significantly commensurate increase in Auger electrons following irradiation with X-rays with energies just above the K-edge or higher. This finding was consistent with earlier studies targeting the K-edge with monochromatic synchrotron X-rays.

Based on these earlier studies, one of the critical issues was the overall and specific energy dependence of X-rays of different energies and their interaction with HZ radiation sensitizers. The primary reason for the use of MeV X-rays for therapy is their greater depth of penetration compared with lower-energy keV radiation. In the present study, we have addressed this issue both theoretically and experimentally. A theoretical framework and numerical simulations have demonstrated that the loss of low-energy X-ray flux as a function of attenuation with depth can be compensated for by their enhanced radiosensitizing effects compared with high-energy X-rays. This potentially could lead to the use of X-ray sources in the 100–250-kV range for radiation therapy of more superficial tumors. The main purpose of our computational study was to delineate the contribution of largely low-energy inner-shell photoionizations, which lead to cell-killing cascades of Auger electrons. This is distinct from the high-energy Compton scattering of photons that are either scattered along the X-ray beam path or may pass through the tumor without interaction. We have designated the energy region between the L and K shells of a HZ atom as the low-energy X-ray (LEX) range, compared with the MV LINAC high-energy X-ray (HEX) range.

Another issue quantitatively addressed in the present report is that the tumoricidal effects of X-irradiation in combination with Pt compounds had been linked to radiosensitization, because the Pt concentrations were very low. This dichotomy was evident in the range of concentrations (mg/ml) employed in theoretical studies to demonstrate radiosensitization, as opposed to the much smaller *in vitro* and *in vivo* concentrations (μg/ml) that were used experimentally to minimize their intrinsic tumoricidal activity and focus on the effects of combining them with X-ray irradiation. However, as shown by Elleaume and her research team [18–21] and Barth and his co-workers [22, 23], there was strong synergism between radiation doses and either cisplatin or carboplatin at therapeutic doses when they were administered intracerebrally (i.c.) by convection-enhanced delivery (CED) to F98 glioma-bearing rats [17–23]. CED completely bypasses the blood–brain barrier (BBB) and maximizes delivery to a brain tumor [24].

As will be discussed in more detail in this report, the LEX range for Pt and Au would correspond to 14–80 keV, which is the spectral peak distribution from LEX 100–250-kV sources. Therapeutic efficacy has been quantified by a numerical model of enhanced radiosensitization by LEX sources compared with HEX radiation with a LINAC using a water and Pt phantom. In order to experimentally validate these models, we carried out *in vitro* experiments using F98 rat glioma cells exposed to either Typ-Pt, which had low *in vitro* cytotoxicity to allow differentiation between chemo- and radiotoxic effects, or carboplatin, a commonly used Pt-based chemotherapeutic agent. These were followed by *in vitro* studies with carboplatin using F98 glioma and B16 melanoma cells due to the unforeseen *in vivo* neurotoxicity of Typ-Pt.

## MATERIALS AND METHODS

### Monte Carlo simulations

The general-purpose Monte Carlo code package Geant4.9.4.p02 and its extension to electromagnetic processes were used to perform the simulations. These followed the algorithm previously developed by us [13], which described Auger photon and electron emissions as a function of X-ray energy. Broadband X-rays were simulated at 10-keV intervals to resolve the energy dependence of DEFs, which was dependent on the X-ray flux. A (15 × 5 × 5)-cm right rectangular water-filled prism, with a 2-cm-wide tumor section delineated 10 cm inside the prism, was used to simulate a tumor surrounded by normal tissue. The tumor Pt concentration was assumed to be 7 mg/ml, homogeneously dispersed in water in a tumor within the phantom, in order to simulate a radiosensitized tumor. X-ray path-lengths and secondary effects, such as ionizations and Auger decays, were tracked and the results were recorded. An X-ray beam originating at 0 cm was tracked for a total length of 15 cm in bins of 0.1 cm (1 mm), and the average energy deposited per photon per bin was recorded. The error for each bin was <1% of the average energy, which also translated into ∼1% of the total error in the phantom. A default cut-off distance of 0.001 mm was used to determine the range of all particles. The DEF in each energy bin was taken by dividing the total energy deposited in a Pt-sensitized tumor by that deposited in a tumor without Pt. In previous Geant4 simulations we did not distinguish between the photoelectric (PE) and scattering components of interaction, but took into account the combined total attenuation of X-rays in the phantom. These simulations were essentially similar to earlier reports using Au as a radio-sensitizer [12, 13], except that Au was replaced with Pt at a concentration of 7 mg/ml.

It should be noted that this HZ concentration was also close to that adopted in several previous Monte Carlo simulations using GNPs [2], as well as *in vivo* studies in tumor-bearing mice [1, 10]. The numerical simulations employed a high sensitizer concentration in order to delineate the difference between activation by 160-kV versus 6-MV X-rays. While the cellular uptake and microdistribution of Pt in our studies was not determined, the numerical computations employed sufficiently large concentrations to elucidate the nature of interactions across a broad range of rapidly varying LEX spectra. For the purpose of these simulations we assumed the theoretical efficacy of activation to be proportional to the Pt concentration, although empirically other important factors such as toxicity, Pt uptake, biological effectiveness, and inhomogeneities in cellular microdistribution could all play important roles.

### Photoionization model of X-ray attenuation

Photoionization or PE absorption of radiation by inner-shell electrons in an HZ atom determines the cascade of high-linear energy transfer (LET) tumoricidal Auger electrons and the accompanying photon emissions via Coster–Kronig and Super-Coster–Kronig transitions [14]. We have constructed a numerical model of photoionization and X-ray attenuation as a function of depth in order to characterize photoionization of the HZ sensitizer at a specified concentration. We have also defined and computed a critical energy, E_c_, where scattering begins to dominate PE absorption or photoionization. The PE component (leading to Auger decays) and the total absorption (including photon scattering) were computed separately. The attenuation coefficients κ, due to photoionization, were convolved over the broadband source distribution *f_bb_(E)* from an X-ray device with potential *V* (kV), up to each depth *d*, to obtain the integrated absorption according to:

(1) $$I\left(d,V\right)\int_{0}^{d}\int_{E_{min}}^{V}\;f_{bb}^{kV}\left(E\right)exp\left(-\rho k_{E}x\right)dEdV$$

Here *ρ* referred to the density of either water or a combination of elemental Pt in water with the two Pt concentrations of either 1 or 7 mg/ml. Since we were interested in only the ‘relative’ behavior of 160-kV versus 6-MV radiation with respect to photoionization, the results were independent of any given concentration *per se*. While Geant4 may also be employed to yield the quantities of interest, the photoionization models presented in this report were computed using a new diagnostic Fortran code XPHOT to complement the Monte Carlo simulations [12]. Furthermore, in order to understand the overall efficacy of LEX versus HEX radiation (in addition to the results reported herein), XPHOT was also designed as a generalized code to study monochromatic localized energy deposition via Kα resonance fluorescence as a function of incident X-ray intensity and HZ concentration [12, 13]). Cross-sections for the primary physical processes, coherent and incoherent (Compton) photon scattering, and PE absorption, were obtained from the XCOM database at the National Institute of Standards and Technology (www.nist.gov) [25]. Although the photoionization algorithm is quite general, we assumed certain representative values and a water plus Pt phantom at a depth *(d) = 15 cm* for the illustrative results presented here. The assumed Pt concentrations were either 1 or 7 mg/ml at a depth of between 10 and 11 cm. The effective range of Auger electrons and the absorbed dose in a water phantom as a function of distance from AuNPs were computed by Leung *et al*. [8]. It was reasonable to assume that the results were equally applicable to Pt, and they showed that the effective range of secondary electrons and the absorbed dose decreased rapidly to small values within 50 µm for 50–250-kV irradiation, and within 1000 µm for the 6-MV irradiation. But it is the low-energy component of the 6-MV source, E ≤∼100 keV, which contributes to photoionization and hence the Auger decays, which therefore are localized to a cellular target of ∼100 µm.

### Synthesis of terpyridine-Pt complex [Pt(typ)(py)](NO_3_)_2_]

The Pt(II) complex [Pt(typ)(py)](NO_3_)_2_, (M.W. = 631.4 Da), designated Typ-Pt, contains the tridentate tpy 2,2′:6′,2″- terpyridine (tpy) complex ligand and one monodentate pyridine (py) ligand, while the nitrate ions are not coordinated to the Pt center and represent counter-ions to the divalent cationic complex. The increased number of bonds (denticity) between typ and Pt results in less reactivity due to typ's inability to dissociate from the central platinum ion, so that only the py ligand can dissociate from the molecule to bind to DNA. However, unlike cisplatin and carboplatin, the DNA binding by Typ-Pt can only take place through a single covalent bond. These features should result in lower overall tumoricidal activity because Typ-Pt is not able to form DNA strand cross-links. Silver nitrate (99.9% Ag) and di-iodo(1,5-cyclooctadiene)Pt(II) 99% were purchased from Strem Chemicals (Newburyport, MA). 2,2′:6′,2″-terpyridine was purchased from Sigma (St Louis, MO), and pyridine was obtained from Mallinckrodt Chemicals (Phillipsburg, NJ). Acetonitrile and acetone were purchased from Fisher (Fisher Scientific Company LLC, PA) and water was deionized using a Barnstead B-pure cartridge water purification system to a resistivity of 18 MΩ (Thermo Scientific, IA). A centrifuge from Cole Parmer (3400 RPM) (IL, USA) and a sonicator from Fisher Scientific (FS30) (Fisher Scientific Company LLC, PA) were used. Electrospray ionization mass spectrometry (ESI-MS) measurements were performed on a MicrOTOF spectrometer (Bruker, Billerica, MA) with electrospray ionization (ESI) equipped with an Agilent 1200 LC (Allied Electronics Inc., Fort Worth, TX) and analyzed using the Bruker Daltonics Data Analysis version 3.4 software (Bruker Corporation, MA). ^1^H NMR spectra were recorded on a 400-MHz Bruker instrument (Bruker Corporation, MA) where s, d and m denote singlet, doublet and multiplet, respectively.

The precursor complex [Pt(typ)(CH_3_CN)](NO_3_)_2_ was synthesized *in situ* according to a published procedure [26]. Briefly summarized, a solution of AgNO_3_ (65.4 mg, 0.385 mmol) in 500 µl acetone/H_2_O (4:1, v:v) was added drop-wise to a suspension of Pt(COD)I_2_ (98.7 mg, 0.177 mmol, COD = cycloocatadiene) in 150 µl H_2_O and 600 µl acetone in a 9 ml vial protected from light with aluminum foil. The mixture was sonicated for 5 min, then centrifuged, and the supernatant was transferred to a new vial, while the precipitate, AgI, was discarded. A solution of 2,2′:6′,2″-terpyridine (34.7 mg, 0.149 mmol, tpy) in 300 µl acetonitrile was added drop-wise to the supernatant, and a bright yellow precipitate formed. The mixture was sonicated for 5 min and then centrifuged. The supernatant was discarded, and 5 ml of pyridine was added to the solid yellow product [Pt(typ)(CH_3_CN)](NO_3_)_2_. The mixture was refluxed with stirring for 2 h, and then excess pyridine was removed by evaporation. The yellow-orange solid was washed with three portions of 1:3 (v:v) acetonitrile/ether and dried with a stream of argon (47.8 mg, 42.8% yield). ^1^H NMR (D_2_O, 400 MHz): δ 7.64 (t, 2 H, *J* = 6.42), 7.75 (d, 2 H, *J* = 5.71 Hz), 7.88 (t, 2 H, *J* = 6.61 Hz), 8.32 (m, 7 H), 8.43 (t, 1 H, *J* = 8.12 Hz), 9.08 (d, 2 H, *J* = 5.17 Hz). ESI-MS *m/z* = 253.6 corresponds to [Pt(typ)(py)]^2+^. The 4-h IC_50_ of this compound was 3.5 μg/ml, and a concentration of 2 μg/ml resulted in 90% survival [26].

### Irradiation procedure

Megavoltage 6-MV X-irradiations were performed using a Siemens Medical Systems (Malvern, PA) LINAC located in the OSU Department of Radiation Oncology. The LINAC dose rate at the position of the cells was 2.2 Gy/min. The F98 glioma [27] and B16 melanoma [28, 29] have been widely used for *in vitro* and *in vivo* studies. Cells were exposed to the Pt compounds in six-well Falcon Multiwell™ plates (Becton Dickinson, Franklin Lakes, NJ) for 4 h, trypsinized, spun down, re-suspended in fresh media, transferred into sterile 2-ml tubes, and then irradiated. Prior to irradiation, the cells were placed in a plastic holder, and water was added to a level of 1-cm depth, which provided back-scatter (required to improve the accuracy of the dose calculation). The dose was calculated from data tables established when the LINAC was commissioned, incorporating the fall-off with distance, attenuation in media, and scatter, both internally in the LINAC and within the apparatus. Irradiations were performed at a source-to-axis distance (SAD) of 100 cm with a field-size of (25 × 25) cm at the isocenter. Tubes containing the cells were placed in an acrylic block drilled specifically for the shape of the tubes. The depth of the tubes' centers from the block edge was 2 cm. The block was setup so that the gantry faced the tubes perpendicularly, and the center of the block was placed at isocenter. Dose was calculated to the tube-center depth (2 cm). It should be noted that the acrylic isn't solid water, but given the current setup there was minimal error by assuming it was water. The 160-kV X-ray irradiations were performed using an RS2000 Small Animal Irradiator (Rad Source Technologies Inc., Suwanee, GA). The irradiation chamber floor was calibrated to deliver 1.13 Gy/min to that height.

### *In vitro* and *in vivo* studies with Typ-Pt and carboplatin

**Fig. 1.**
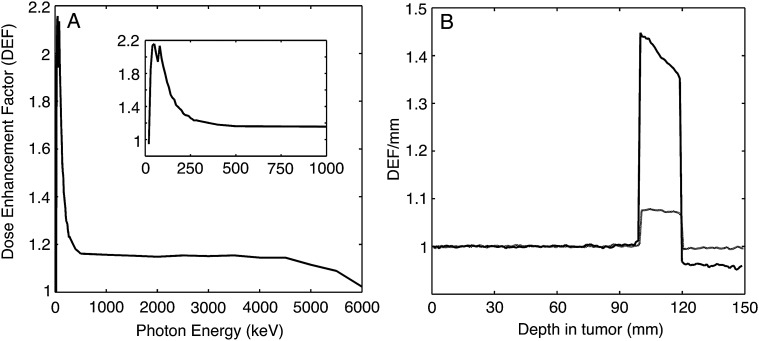
(**A**) DEFs from 20 keV to 6000 keV (6 MeV) X-rays as a function of energy. The DEF peaked in the LEX region and up to 500 keV. (**B**) DEFs as a function of depth in the phantom for 160 kV (black, higher DEF) and 6 MV (grey, lower DEF) with a Pt-sensitized tumor at a depth of 100–120 mm (10–12 cm).

Initially, *in vitro* studies were carried out with Typ-Pt. Clonogenic assays were performed by plating F98 glioma cells into Falcon Multiwell™ plates, incubating them overnight, and then exposing them to 0–4 µg/ml of Typ-Pt. After a 4-h incubation period, the cells were trypsinized, centrifuged, re-suspended in Pt-free medium and transferred to 1.5-ml microcentrifuge tubes. They then were irradiated with a 7-Gy dose of either 160-kV X-rays or 6-MV photons, as described above. Surviving fractions (SFs) were calculated by counting cell colonies composed of >50 cells under a dissecting microscope. The SFs of irradiated cells were normalized to highlight the effects of radiosensitization. A second study was performed to evaluate the effects of varying the X-ray dose. Cells were treated with a non-cytotoxic concentration of Typ-Pt (2 μg/ml) and then irradiated with either 160-kV or 6-MV X-rays at doses of 0, 1, 2.5, 5, 10 and 15 Gy. Since the purpose of performing the Typ-Pt study was to compare the relative reductions in SFs of sensitized versus unsensitized cells, the ratios of the surviving fractions (SF ratios) at each X-ray dose were computed for each treatment group. These ratios were calculated by dividing the SF of the cells that had received X-irradiation alone by that of cells irradiated following exposure to Typ-Pt, resulting in the relative enhancement in cell killing with sensitization. This approach was used to demonstrate the increasing enhancement ratio as a function of the X-ray dose in LEX sensitization compared with near constancy with HEX with very little sensitization for the latter. 160 kV and 6 MV cell-killing enhancement ratios vs unsensitized cells are directly compared, albeit with larger uncertainties than SFs for a given X-ray source. However, it should be noted that if these were computed in terms of the ratios of doses leading to SFs, then the LEX enhancement would be much smaller and in agreement with the theoretical DEFs shown in Fig. 1 (see Discussion).

Unexpectedly, Typ-Pt was found to be highly neurotoxic when administered i.c. by CED to non-tumor-bearing Fischer rats. This technique completely bypasses the BBB, maximizes delivery of the therapeutic agent to the brain and minimizes uptake by extracranial organs and blood [24]. Therefore, low doses of carboplatin were evaluated as an *in vitro* radiation sensitizer using the same methods as have been described in our studies with Typ-Pt. Cells were treated with 0–5 μg/ml of carboplatin and then irradiated with 7 Gy of 160-kV X-rays. Data from a similar experiment performed with 6-MV X-rays, which were reported previously [22], have been included for comparison. A second experiment was performed by exposing cells to 0, 1.0 and 2.5 μg/ml of carboplatin and then irradiating them with the same 0–15-Gy doses of 160-kV X-rays. The concentration of Pt in each cell was determined by assuming a cellular uptake of the sensitizer that was equal to the concentration in the culture media. The cell density was assumed to be 10^9^ cells/ml, and the amount of sensitizer in the cell was determined based on the molecular weights of Typ-Pt (631.4 Da) and carboplatin (371.2 Da). The ratios were calculated for both the 160-kV and 6-MV irradiations groups to highlight the difference in survival of radiosensitized versus unsensitized cells after irradiation with either LEX or HEX. The survival at high dose rates was very small. Therefore the ratio of the SFs of sensitized versus unsensitized cells (rather than the absolute SFs) were presented to highlight this difference.

To further support the data obtained with the F98 glioma cell line, as well as to test the applicability of this form of radiation therapy to another type of cancer, the experiments were repeated using carboplatin and the murine B16 melanoma cell line. Clonogenic assays were performed using the same procedure as that described for the F98 glioma cells, as described above. B16 cells first were exposed to varying concentrations of carboplatin (1–7 μg/ml) and then irradiated with an X-ray dose of 3 Gy to better delineate the difference in survival. For the second part of the study, the cells were incubated with 2.5 μg/ml of carboplatin for 4 h and then irradiated with varying doses of either 6-MV photons or 160-kV X-rays (0–15 Gy) to obtain an independent verification of the data obtained with Typ-Pt and F98 cells. The SF ratios for each X-ray dose were computed as described previously.

### Neurotoxicologic studies of Typ-Pt following convection-enhanced delivery to Fischer rats

All animal studies were carried out in accordance with National Research Council guidelines [30] and approved by the Institutional Laboratory Animal Care and Use Committee of the Ohio State University. A dose de-escalation study was carried out with 200, 100, 50, 25 or 12.5 µg of Typ-Pt, administered i.c. by CED to non-tumor-bearing Fischer rats (Charles River Laboratories International Inc., Wilmington, MA), as previously described [22, 23]. Except at the lowest dose, all rats that received Typ-Pt i.c. demonstrated clinical evidence of neurotoxicity and were euthanized once the effects of anesthesia had worn off. As described in the Results, the brains of all of the euthanized rats were removed, fixed in 10% buffered formalin, and then sectioned coronally at 2-mm intervals and processed for histopathologic examination, which included staining of the sections with hematoxylin and eosin (H&E).

### Statistical analysis

Each datapoint in the SF plots consisted of triplicate plates, and the means and standard deviations (SDs) were computed for the SFs. These were plotted in Figs 3 and 4. Data were normalized by natural log transformation to minimize the variations. The linear–quadratic (LQ) model was used to fit the data.

Student's *t*-test was used for the experiments with two groups involved, and analysis of variance (ANOVA) was used for the experiments with multiple groups involved. Holm's method was applied to adjust multiplicities to control the family-wise error rate at 0.05. SAS 9.3 software was used for analysis (SAS Inc., Cary, NC).

## RESULTS

### Monte Carlo simulations

Results for the DEFs from numerical simulations using Geant4 corresponding to 160-kV and 6-MV spectral distributions and Pt homogeneously dispersed in a water-containing phantom are shown in Fig. 1A. The DEFs peaked at ∼80 keV, which was close to the Pt K-edge (78.4 keV). In addition, values >2.0 were found in the LEX energy range of 40–80 keV, between the L and the K edges of Pt. The values for the DEF leveled off after ∼200 keV and approached an asymptotic value at slightly greater than unity. For 6-MV photons, there was negligible radiosensitization with Pt for X-rays with energies above the LEX range. Most importantly, the average DEF over the entire Pt-containing tumor volume was 1.81 following irradiation with 160-kV X-rays compared with 1.14 for 6-MV photons, suggesting that little radiosensitization occurred for much of the 6-MV spectrum. In addition, there was no drop in the X-ray flux of the latter (Fig. 1B) after the tumor section of the phantom, indicating that increased absorption only occurred with incident LEX.

### Radiation absorption and scattering: photoionization models

**Fig. 2.**
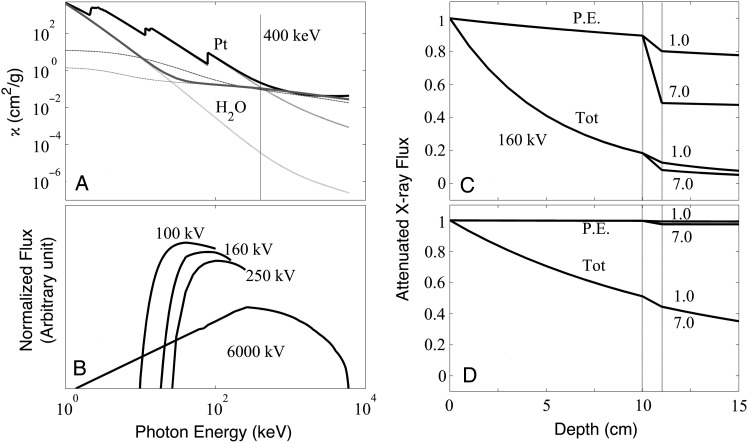
(**A**) Total attenuation coefficients including Compton scattering (solid line) of H_2_O (dark grey) and Pt (black), photoelectric (PE) components (grey lines which minimally decrease as a function of energy) and scattering components (grey lines which show a significant decrease with increasing energy). The scattering component of Pt dominated PE for E > 400 keV; (**B**) normalized radiation ﬂuxes from low-energy sources 100 kV, 160 kV and 250 kV, and the high-energy 6000-kV (6-MV) LINAC photons. (**C**) PE and total X-ray flux attenuation, including Compton scattering by a generalized 1-cm (water + Pt) phantom from a 160-kV source; (**D**) 6000-kV (6-MV) source. The fluxes in (C) and (D) have been calculated using normalized profiles in (B) with Pt concentrations 1 mg/ml (1 µg/µl) and 7 mg/ml (7 µg/µl) located in the phantom at a depth of 10–11 cm.

Whereas the LEX enhancement in DEFs described in the preceding section corresponded to the total radiation flux administered to the sample, we also independently computed the contribution to radiosensitization from the PE absorption component *vis-à-vis* the photon scattering component. The PE absorption via photoionization was compared with the total X-ray absorption as a function of depth in the (H_2_O + Pt) phantoms. The total attenuation coefficients (κs) including photon scattering with the PE cross-sections of Pt and water, are shown in Fig. 2A. For Pt dispersed homogeneously in water, the critical energy E_c_ occurred at ∼400 keV. Photoionization of water was negligible at these high energies. The efficiency of Pt as a radiation sensitizer rapidly decreased beyond the K-edge (78.4 keV) as the photon scattering component became increasingly dominant. The total attenuation coefficient approached a constant asymptotic value above E_c_. There was no significant increase in energy deposition for radiosensitized phantoms irradiated with X-rays > E_c_ (∼400 kV).

A comparison of the 160-kV LEX and the 6-MV HEX spectra is shown in Fig. 2B. Although the effective spectra from 100–250-kV sources peaked in the LEX range, only a small fraction of the 6-MV spectrum was between the L and K edges, where photoionization cross-sections were the largest. This lower-energy component of MV LINACs with photons of ∼100 keV would still be effective for radiosensitization. This is the most likely explanation for previous simulations that showed some dose enhancement with GNPs irradiated with 6-MV X-rays using LINACs [10]. However, since the absolute number of photons in this range was small, as evident from the normalized output (Fig. 2B), HEX radiosensitization was not effective.

Next, using results from the computer program XPHOT, the photoionization or PE component, which was responsible for Auger decays and cell killing, was isolated from the total X-ray flux. A detailed analysis of the LEX and HEX sources with 160-kV and 6-MV output spectra are shown in Fig. 2C and D. Radiosensitization was evident at two different Pt concentrations at a tumor at a depth of 10 cm within the phantom. Several important conclusions can be drawn from these observations. First, irradiation with 6-MV photons resulted in virtually no additional photoionization and therefore would not have caused an enhancement of high-LET Auger electron emission via intra-shell or inter-shell electronic cascades, driven by Coster–Kronig or Super-Coster–Kronig transitions, and a general theoretical construct recently has been developed (S. Lim and A.K. Pradhan, submitted for publication). Second, a large PE differential occurred with 160-kV X-rays compared with 6-MV photons between 1 and 7 mg/ml of Pt, with the latter being indistinguishable between the two concentrations, which demonstrated no difference in PE. It was also found that 100–250 kV X-ray sources producing output spectra in the LEX range had similar behavior.

Using Eq. (1) and flux distributions in Fig. 1, we calculated the ‘ratio’ of PE attenuation between LEX 160 kV and HEX 6 MV to be nearly a factor 18 in favor of the LEX, due to a change in concentrations from 1 mg/ml to 7 mg/ml. In other words, photoionization from LEX 100–250-kV sources could be well over an order of magnitude more effective in producing Auger electrons from Pt than 6-MV photons. More of the attenuated X-ray flux got through at high rather than at low energies. Almost 50% of the attenuated flux from the 6-MV photons penetrated the phantom at a tumor depth of 10 cm compared with 18% from the 160-kV broadband source. However, the reduction was much smaller by factors of 2 and 3, respectively, than the theoretically possible enhancement factors due to radiosensitization of up to an order of magnitude, as demonstrated in Fig. 2.

### *In vitro* studies with Typ-Pt and carboplatin

**Fig. 3.**
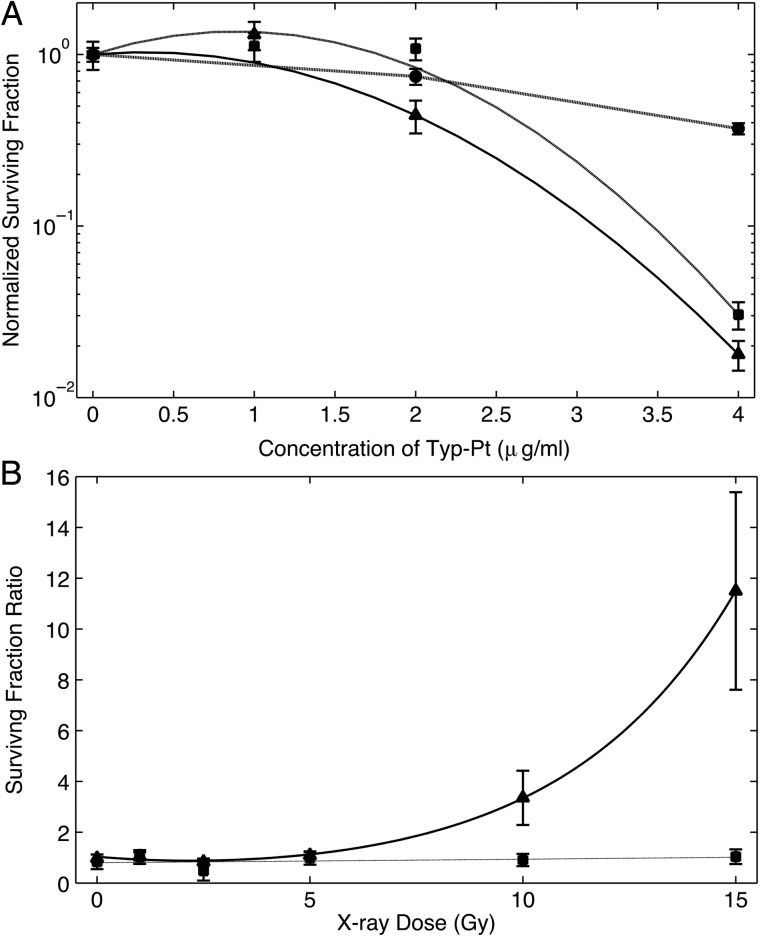
(**A**) Clonogenic survival of F98 glioma cells following exposure to non-therapeutic concentrations Typ-Pt, with or without radiation. Cells were irradiated with either 0 or 7 Gy: 0 Gy (closed circles), 7 Gy 6-MV photons (closed squares), 7 Gy 160-kV X-rays (closed trianges). Beginning at a Typ-Pt dose of 2 µg/ml, cells irradiated with 160-kV X-rays had significantly reduced survival compared to those irradiated with 6-MV photons (*P* = 0.0002 for 2 µg/ml, *P* = 0.002 for 4 µg/ml). Surviving fractions at 0 µg/ml Typ-Pt were normalized to 1 to show clearly the effects of increasing the sensitizer dose. (**B**) Surviving fraction ratio (SF ratio), or enhancement in cell killing, of sensitized versus unsensitized cells with Typ-Pt and either 6-MV photons (closed squares, grey line) or 160-kV X-rays (closed triangles, black line). Based on a comparison with unsensitized cells irradiated with 160-kV X-rays, the relative increase in SF ratio of cells treated with Typ-Pt was significant, starting from a dose of 10 Gy (*P* < 0.0001), whereas no difference was seen for cells treated with 6-MV photons. The normalization and fitting procedure for different sets of data are discussed in the text.

Cells exposed to cytotoxic concentrations of Typ-Pt varying from non- to weakly cytotoxic showed a marked decrease in SFs after irradiation with a 7-Gy X-ray dose (Fig. 3A). The results demonstrated that as the Pt concentration increased, significantly greater cell death (*P* < 0.005) occurred using 160-kV compared with 6-MV X-rays. This was most probably due to increased attenuation of PE absorption by Pt atoms. The SFs of the irradiated cells were normalized to 1 to highlight the increased cytotoxic effects of X-rays when used with HZ radiosensitization. Figure 3B shows the survival ratios of F98 cells treated with Typ-Pt and X-rays by plotting the relative increase in the SF ratios of unsensitized versus sensitized cells that had been exposed to either 160-kV or 6-MV X-rays for a range of doses. Beginning at 10 Gy, radiosensitized cells irradiated with 160-kV X-rays, showed a significant increase (*P* < 0.0001) in the SF ratio compared with those exposed to X-rays alone. The radiosensitized cells irradiated with 6-MV photons did not show a similar increase in SFs at any X-ray dose. The concentration of 2 μg/ml of Typ-Pt, corresponding to a theoretical uptake of 1.91 × 10^6^ Pt atoms per cell, was weakly cytotoxic, suggesting that the increase in cell death was due to radiosensitization by Pt.

In agreement with both the calculations of PE versus total absorption using XPHOT, and the Monte Carlo simulations for DEFs using Geant4, F98 cells irradiated with 160-kV X-rays had lower SFs relative to the cells treated with 6-MV photons by up to a factor of 10. The most probable reason for this increase in sensitization was that the coefficients for PE absorption by Pt were considerably higher than the total absorption by water at keV energies in the LEX range. This resulted in more ionizations and consequently enhanced production of low-energy Auger electrons that act like high-LET radiation. While HEX-MV photons will interact with a HZ radiosensitizer, the number of high-LET electrons emitted would be marginal compared with those resulting from LEX sources.

**Fig. 4.**
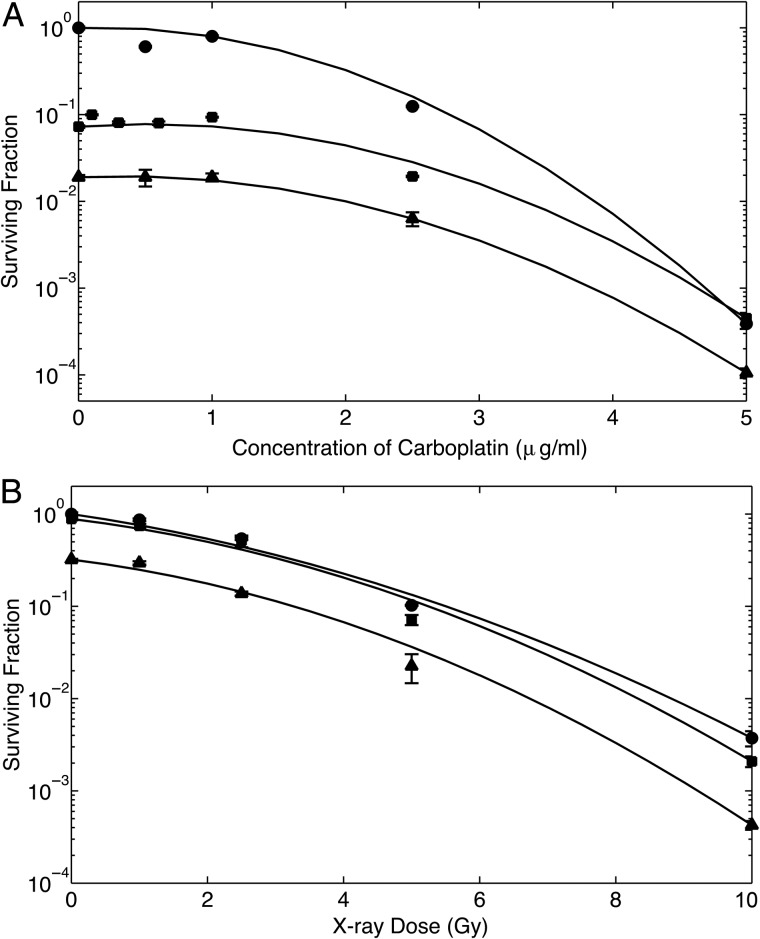
Clonogenic survival of F98 glioma cells following exposure to carboplatin with or without irradiation. (**A**) Unirradiated cells (closed circles), irradiated with 7 Gy of either 6-MV photons (closed squares) [22] or 160-kV X-rays (closed triangles). The decrease in SFs between sensitized cells irradiated with 160 kV versus 6 MV was significant (*P* < 0.05) at all doses of carboplatin. The increased radiotoxicity of 160 kV relative to 6 MV at 0 µg/ml carboplatin was expected and is due to the higher LET of LEX. This is discussed further in the text. No normalization was made with respect to sensitizer dose because the mechanism of radiosensitization in this case is via inhibition of DNA repair, rather than increased electron emissions. (**B**) Cells irradiated with varying doses of 160-kV X-rays at three different carboplatin concentrations: 0 (closed circles), 1 (closed squares) and 2.5 µg/ml (closed triangles). Treatment with 2.5 µg/ml of carboplatin resulted in a significant decrease in SF (*P* < 0.0005) across all X-ray doses. Error bars are not visible on some datapoints due to the size of the marker.

Similar to the results obtained with Typ-Pt-treated F98 cells, those that had been exposed to increasing concentrations of carboplatin and then irradiated with 7 Gy of 160-kV X-rays consistently showed significantly decreased SFs (*P* < 0.05) compared with those irradiated with 6-MV photons (data from Yang *et al*. [22]) for the same carboplatin concentration. The decrease in SFs was more pronounced for all carboplatin concentrations, and was greater than that seen with Typ-Pt. This may have been due to the increased intrinsic cytotoxicity of carboplatin (Fig. 4A). Even without radiosensitization (i.e. at 0 µg/ml carboplatin), irradiation with an LEX source results in decreased SFs. The dependence of SFs as a function of the 160-kV X-ray dose for three different carboplatin concentrations (0, 1 and 2.5 µg/ml) is shown in Fig. 4B. While there appeared to be a decrease in SFs for sensitized versus non-sensitized cells for those treated with 1 µg/ml of carboplatin (1.62 × 10^6^ atoms of Pt per cell) after an X-ray dose of 5 Gy. This difference was not significant. For cells sensitized with 2.5 µg/ml of carboplatin (4.05 × 10^6^ atoms of Pt per cell), the SFs showed a marked decrease relative to the non-sensitized cells (*P* < 0.0005). While this is qualitatively consistent with our hypothesis, our quantitative results require a more nuanced interpretation. Possible reasons for these observations are explored in the Discussion section.

**Fig. 5.**
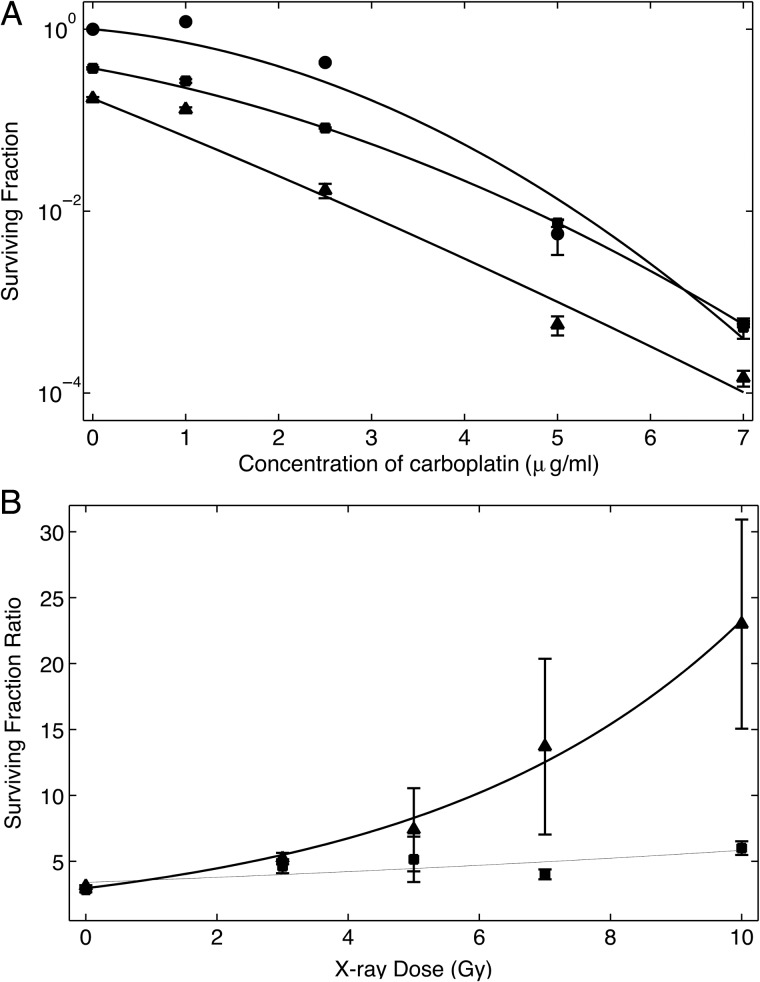
Clonogenic survival of B16 melanoma cells following exposure to carboplatin, with or without irradiation. (**A**) Unirradiated cells (closed circles), irradiated with 3 Gy of either 6-MV photons (closed squares) [21] or 160-kV X-rays (closed triangles). The decrease in SFs between sensitized cells irradiated with 160 kV versus 6-MV photons was significant (*P* < 0.01) at all doses of carboplatin. The reductions in SFs with 160-kV X-rays relative to 6-MV photons at 0 µg/ml of carboplatin was similar to that seen with F98 cells and again can be attributed to impaired DNA repair, as has been discussed in the text. (**B**) Relative increase in the surviving fraction ratio of unsensitized cells versus sensitized cells, indicating the enhancement in cell killing. The plots show the ratio of carboplatin-sensitized cells irradiated with varying doses of either 6-MV photons (closed squares, grey line) or 160-kV X-rays (closed triangles, black line). While irradiation with both radiation sources resulted in a decrease in SFs relative to unsensitized cells, the reductions in SFs for cells irradiated with 160-kV X-rays were greater than those irradiated with 6-MV photons. The large error bars were due to the very low survival for sensitized cells irradiated with 160-kV X-rays.

The data obtained using F98 glioma cells were also confirmed using murine B16 melanoma cells. B16 cells irradiated with 3 Gy of 160-kV X-rays consistently show significantly decreased survival (*P* < 0.005) for all corresponding doses of carboplatin compared with those treated with 6-MV X-rays (Fig. 5A). As with the F98 cells, the decrease in SFs, even without radiosensitization (0 µg/ml carboplatin), demonstrated cell killing with LEX sources. The differences in clonogenic survival with respect to the radiation doses were studied in the same manner as that which was used with Typ-Pt and F98 cells. Similar to Fig. 3B, Fig. 5B shows the ratio of the SF of unsensitized versus sensitized cells. Sensitized cells that were irradiated with 160-kV X-rays exhibited a significantly higher (*P* < 0.01) SF ratio relative to unsensitized cells compared with those irradiated with 6 MV. Unlike the data obtained with Typ-Pt and F98 glioma cells, however, the SF ratio for cells irradiated with 6 MV was not close to unity. This will be explored in the Discussion.

### Neurotoxicologic studies

The brains of rats that received 100 and 200 µg of Typ-Pt i.c. by CED showed cerebral edema, as evidenced by a flattening of the cortical surfaces and considerable congestion of the intracerebral penetrating vessels. The brains of rats that received 200 µg of Typ-Pt also showed intracerebral hemorrhage and necrosis along the path of the needle track (Fig. 5A) and subarachnoid hemorrhage and congestion and bleeding into the choroid plexus. The brains of rats that received 100 µg of Typ-Pt exhibited subarachnoid hemorrhage and scattered clusters of small, red, shrunken neurons (Fig. 5B), and following i.c. CED of 50 µg of Typ-Pt there were foci of multiple small hemorrhages in the immediate subcortical region and congestion of small subarachnoid vessels (Fig. 5C). All of these changes represented acute effects of Typ-Pt and could have been due to the molecule itself or possibly the NO_3_^2−^ anion. However, based on the clinical and gross neuropathologic findings, it was decided that, in a future *in vivo* study, the radiosensitizing properties of carboplatin at a non-therapeutic dose of 2.5 µg in combination with either 160-kV X-rays or 6-MV photons would be evaluated.

## DISCUSSION

Quantitative simulations and *in vitro* experiments have demonstrated that 160-kV X-ray sources were more effective compared with 6-MV photons in reducing the SFs of F98 glioma cells following their exposure to either Typ-Pt or carboplatin. These observations supported our hypothesis that cell survival was dependent on the energy of the X-rays and that radiosensitization was more effective when cells were irradiated with X-rays spanning the entire region between the L- and K-shells of the HZ sensitizer. Although the amounts of Pt that were used for these simulations were high, in reality they would have been equivalent to 1 µg or 7 µg/µl of carboplatin, and as previously reported by [22], a dose of 20 µg in 10 µl was administered i.c. by CED to F98 glioma-bearing rats.

In our computational studies we have shown that PE absorption decreased with energy, while coherent scattering and the much larger incoherent Compton scattering cross-sections of high-energy photons increased, even in the presence of the radiosensitizer. The scattering cross-sections eventually were dominant beyond the critical energy, E_c_. For Pt compounds, the E_c_ was ∼400 keV. Even with the loss of flux due to attenuation as a function of depth, the radiation dose delivered by LEX X-rays from a 160-kV source would have been approximately two to three times greater than that from 6-MV photons. This would have more than compensated for a loss in dose due to attenuation as a function of depth. Therefore, it was possible to obtain a significant dose enhancement factor via photoionization, leading to increased Auger electron production and cell killing using lower-energy X-rays. The majority of the high-energy photons were scattered along the path to the tumor or passed through it with or without a HZ radiosensitizer, presumably damaging normal tissues in the process. In contrast, lower-energy X-rays with a broad peak distribution can target both the L and K shell electrons of the HZ sensitizer and would be more effective in delivering a tumoricidal dose and sparing the intervening normal tissues. Since the K and L shell energies of Au are only slightly higher than that of Pt, the findings presented here should be equally applicable to GNPs. However, it must be emphasized that the enhanced *in vitro* and *in vivo* tumoricidal effects of the combination of X-irradiation and a Pt sensitizer may also be due to completely different radiobiologicalmechanisms [31–35]. As has been reported by Elleaume and her research team [18–21] and Barth and his co-workers [22, 23], when a tumoricidal dose of carboplatin was combined with X-irradiation, there was very strong synergy between the two [22]. The formation of Pt adducts with nucleophilic sites in DNA molecules can cause cell cycle arrest in G_1_ and G_0_ [31] and can activate apoptotic pathways [32]. This can interfere with the repair of radiation-induced damage and may explain the interaction between high enough concentrations of cisplatin or carboplatin and ionizing irradiation, thereby interfering with DNA repair [33–35]. In our study, however, we attempted to separate out the effects of radiosensitization via increased electron production from inhibition of DNA repair by using non-chemotherapeutic concentrations of carboplatin that would form minimal DNA cross-linkages.

**Fig. 6.**
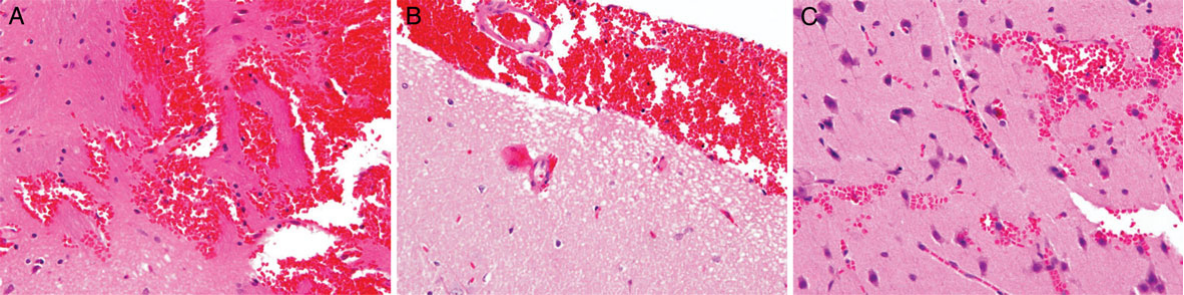
Neuropathologic changes associated with i.c. convection-enhanced delivery of Typ-Pt into the right cerebral hemisphere of non-tumor-bearing Fischer rats. (**A**) 200 µg. Intracerebral hemorrhage and necrosis along the path of the needle track. (**B**) 100 µg. Subarachnoid hemorrhage associated with congestion of intracerebral vessels. (**C**) 50 µg. Focus of multiple small hemorrhages in the immediate subcortical region. H&E-stained sections. Magnification: (A) and (B) × 200; (C) ×400.

Typ-Pt was synthesized in order to have a Pt compound that had lower intrinsic cytotoxicity for F98 cells (IC_50_ = 3.5 µg/ml) compared with carboplatin (IC_50_ = 0.437 µg/ml [26]). However, as shown in Fig. 6, i.c. CED of this compound to non-tumor-bearing Fischer rats proved to be highly neurotoxic. Nevertheless, the SFs of cells treated with either Typ-Pt or carboplatin and then irradiated with 7 or 3 Gy of X-rays shared a striking similarity. In both cases, there was a greater decrease in SFs of radiosensitized cells irradiated with 160-kV X-rays compared with those treated with 6-MV photons (Figs 3A, 4A and 5A). The normalized cell survival plots for non-tumoricidal doses of Typ-Pt are shown in Fig. 3A. This compound, based on chemical considerations, was incapable of forming DNA-adducts and thereby interfere with DNA repair mechanisms. The low cytotoxicity of typ-Pt at concentrations between 0 and 2 μg/ml strongly suggested that the decrease in SFs relative to HEX was due to increased electron emissions via interaction of LEX with Pt. In Figs 4A and 5A, however, any normalization with respect to the sensitizer dose, as illustrated in Fig. 3A, would produce minimal enhancement. This suggests that the intrinsic cytotoxicity and inhibition of DNA repair by carboplatin was the predominant effect. The decreased survival of unsensitized F98 B16 cells, as shown in the 0 µg/ml carboplatin concentration (Figs 4A and 5A), and the non-normalized SFs for Typ-Pt at 0 µg/ml (data not shown) for cells that were irradiated with LEX compared with HEX, could be explained by the difference in fluence between both sources as a function of proton energy. LEX have a considerable fluence at lower energies where the PE cross-sections of water are non-negligible (Fig. 2A and B), thereby increasing electron emissions from water and causing more cell death relative to HEX, even without any radiosensitizer. While irradiation with LEX would cause greater electron emissions from Pt and water and result in decreased cell survival, the mechanism of radiosensitization with carboplatin, compared with Typ-Pt, most likely was due to the inhibition of DNA repair rather than increased electron emissions.

Heavy elements such as Pt enhance this process due to their higher PE absorption coefficients at LEX energies and the greater number of electrons bound per atom. By using X-rays with energies that can target both the L and K shell electrons, we were able to concentrate the dose in the DEF region containing the two peaks (Fig. 1A) and activate the PE absorption of Pt in the cells sensitized with either Typ-Pt or carboplatin. However, due to the low concentrations of the sensitizers, in order to minimize their intrinsic cytotoxicity, this effect was small. This was evident with Typ-Pt because the concentrations used were non-toxic (Fig. 3A and B), which in turn allowed us to isolate radiosensitization as a function of increased electron emissions rather than the inhibition of DNA repair. In this case, the combination of LEX with Pt enhanced the production of high-LET Auger electrons from HZ atoms compared with HEX or monochromatic X-rays at the K edge of Pt. This resulted in decreased SFs for radiosensitized F98 cells following irradiation with 160-kV X-rays (Fig. 3A and B). For B16 cells pre-treated with carboplatin (Fig. 5B), the enhancement of cell-killing in sensitized cells irradiated with varying doses of 160-kV X-rays was most likely due to decreased DNA repair [31–33] and increased electron emissions. On the other hand, the energy spectrum of 6-MV photons produced by a LINAC was largely concentrated in the high range above the E_c_ of Pt at 400 keV. This suggested that much of the dose resulted in photon scattering instead of PE. This would explain the unchanged relative survival of sensitized F98 cells after irradiation with 6-MV photons versus cells exposed to radiation alone (Fig. 3B). The slight decrease in the relative SFs shown by the increase in the SF ratios for B16 cells irradiated with 6-MV photons (Fig. 5B) most likely also was due to reduced DNA repair [31]. It is noteworthy that the relative increase in SF ratios remained fairly small and linear for all radiation doses, which further implies that the increase in cell damage due to increasing electron emissions was minimal.

The *in vitro* studies defined X-ray radiosensitization using Pt as a HZ sensitizer, which was highly energy dependent. For example, it is known that targeting the K-edge *per se* by monochromatic synchrotron X-rays does not lead to significantly enhanced Auger emissions and contributes minimally to radiosensitization [24]. However, recently it has been reported that the efficacy of 78.8-keV synchrotron X-rays directed just above the Pt K-edge was equivalent to 6-MV broadband X-ray irradiation [17]. This can be explained by examining both the detailed photoabsorption coefficients and corresponding flux variation with energy, as shown in Fig. 2A. The mass attenuation coefficient at the Pt K-edge fell to ∼9 cm^2^/g, compared with the Pt L-edge, where it was 190 cm^2^/g, ∼20 times higher. Since the 100–250 keV LEX sources have a broad peak distribution in the K-L energy range, the integrated effect using Pt as a radiosensitizer was much greater than at the K-edge alone. Although the K-edge attenuation coefficient was much higher than the coefficients at MeV energies from a HEX 6-MV source, photoabsorption at the K-edge from a monochromatic source remained small compared with that of broadband LEX sources. An important caveat, however, is that a relatively high concentration of the radiosensitizer would be required to see any therapeutically significant differences in survival, even with a broad spectrum LEX source.

For F98 cells treated with a minimally cytotoxic dose of carboplatin (1 μg/ml), we found a slight, although not statistically significant, difference in SFs at all X-ray doses relative to unsensitized cells (Fig. 4B). In contrast, a significant decrease (*P* < 0.005) in SF was observed with Typ-Pt at a non-cytotoxic dose of 2 μg/ml (Fig. 3A). It should be noted that no significant decrease in the SF was seen with a Typ-Pt concentration of 1 μg/ml for cells irradiated with 7 Gy of 160-kV X-rays. This suggested that a certain threshold concentration of the sensitizer was required for radiosensitization, regardless of whether this is due to increased electron emissions or inhibition of DNA repair, as has been commented on by Elleaume *et al*. [36]. The difference in SFs for F98 cells sensitized with a non-toxic 2 µg/ml dose of Typ-Pt and then irradiated with 7 Gy of either 6-MV or 160-kV X-rays was significant (*P* = 0.0002). This strongly suggested that it was radiosensitization by Pt that caused the decrease in cell survival rather than the intrinsic cytotoxicity of the sensitizer used in combination with LEX. Although most of the enhancement in radiosensitization from the 160-kV source occurs from X-rays below the K-edge, it follows that the corresponding cell survival due to Pt Auger decays in proximity to cell nuclei versus cytoplasm [37] was higher with 6-MV photons.

We found a large increase in cell death at all X-ray doses for a carboplatin concentration of 2.5 μg/ml (Figs 4B and 5B). The calculated number of Pt atoms per cell for all treatments were within the same order of magnitude, which does not suggest that the decrease in SFs for cells treated with 2.5 μg/ml carboplatin only was due to HZ radiosensitization. Instead, these results suggest that at a non-cytotoxic concentration of the sensitizer, a decrease in the SFs of radiosensitized versus non-sensitized cells would only be seen after either a minimum threshold dose of X-rays was reached or a critical number of the HZ atoms was present for radiosensitization to occur at lower X-ray doses. This second possibility may explain why a very high dose of GNPs had to be used in previous studies [1, 4, 10] in order to obtain a significant decrease in tumor size with a murine tumor model. One final comment should be made about the *in vivo* biological significance of reducing the SF of F98 glioma cells by one log unit. As has been previously reported by Yang, Barth *et al*. [38], this would have had a very modest effect in increasing the mean survival time of F98 glioma bearing rats.

## CONCLUSION

The present study has extended the findings on the radiosensitizing properties of HZ agents such as GNPs reported by other investigators [1–4, 7, 8, 10] in several ways. First, a quantitative analysis of photoionization versus photon scattering of interaction of X-rays with HZ elements generally confirmed the efficacy of LEX versus HEX radiation using Pt compounds. Second, unlike previous studies, our study has separated the effects of radiosensitization and cytotoxicity by employing non-toxic concentrations of Pt-compounds. Third, in order to reduce the systemic toxicity of Pt-based chemotherapeutic agents such as cisplatin and carboplatin, another Pt-compound, Typ-Pt, was synthesized because of its reduced *in vitro* cytotoxicity. Although *in vitro* experiments demonstrated radiosensitization as expected, i.c. administration of Typ-Pt was highly neurotoxic in non-tumor bearing Fischer rats and therefore precluded further studies with this compound. Fourth, our theoretical model was supported by *in vitro* studies in which the SFs of F98 glioma cells treated with Typ-Pt and irradiated with 160-kV rays showed a decreasing trend with radiation dose compared with a marginal decrease in SFs of cells irradiated with 6-MV photons. The F98 data were concordant with those obtained with murine B16 melanoma cells using carboplatin as the sensitizer. Finally, theoretical simulations have shown that the enhancement of up to an order of magnitude using HZ sensitizers and lower-energy X-rays may more than compensate for the 2–3-fold loss of flux with depth, even with tumors at depths of 10 cm. However, the interdependence between the X-ray dose and the tumoricidal activity of the HZ sensitizer will be investigated in more detail in future studies.

## FUNDING

This research was supported in part by a Large Interdisciplinary Grant from the Ohio State University (SNL, AKP, SN), Voices Against Brain Cancer, and a donation from the Kevin J. Mullin Memorial Fund for Brain Tumor Research. Funding to pay the Open Access publication charges for this article was provided by internal funds from the Departments of Pathology, Chemistry and Astronomy of The Ohio State University.
